# Swarm shepherding using bearing-only measurements

**DOI:** 10.1098/rsta.2024.0145

**Published:** 2025-01-30

**Authors:** Aiyi Li, Masaki Ogura, Naoki Wakamiya

**Affiliations:** ^1^ Department of Bioinformatic Engineering, Graduate School of Information Science and Technology, Osaka University, Suita, Osaka 565-0871, Japan; ^2^ Graduate School of Advanced Science and Engineering, Hiroshima University, Higashi-Hiroshima, Hiroshima 739-8521, Japan

**Keywords:** swarm systems, shepherding control, bearing-only measurements, nonlinear dynamics

## Abstract

Drawing inspiration from natural herding behaviours, shepherding provides a method for swarm guidance that utilizes steering agents and can be applied in biological and robotics systems at various scales. However, while most shepherding research has relied on the precise sensing capabilities of steering agents, these assumptions do not necessarily hold in real-world tasks. To fill in the gap between practice and literature, in this study, we demonstrate that swarm shepherding can be achieved via bearing-only measurements, and explore the minimum amount of information required. We initially formulate our algorithm for a single agent and subsequently expand its application to accommodate multiple agents, incorporating strategies tailored for herding multiple swarms. Numerical simulations show the effectiveness of the algorithm under various initial placements and configurations. The minimum amount of information required by the proposed algorithm for successful shepherding, i.e. a moderate angular accuracy for the steering agents and limited communication between them, is also determined. Our proposed bearing-only algorithm offers crucial insights into swarm dynamics, which may have applications across a variety of domains, such as agriculture and search and rescue.

This article is part of the theme issue ‘The road forward with swarm systems’.

## 1. Introduction

Self-organized group formation and collective motion in the form of swarms are hallmarks of living systems over a wide range of length scales, from microorganisms to fish schools, bird flocks and animal herds [1–4]. These swarm behaviours emerge without centralized coordination and are instead influenced by the interactions among individual group members [5–7]. The study of swarm behaviours has attracted interest in the field of biology and has inspired the development of artificial systems that utilize numerous agents to perform intelligent tasks in the fields of swarm robotics [8] and microrobot systems [9,10].

In many circumstances, attempting to manipulate swarm behaviours or movements directly can be difficult. Therefore, to overcome this difficulty, control over the swarm can be exerted via another class of agents, called steering agents [11,12]. A known paradigmatic example of swarm control is the shepherding problem [13,14], which is inspired by the predatory behaviour of sheepdogs on sheep. In this field of study, researchers focus on designing algorithms for shepherd-like steering agents to guide sheep-like passive agents towards a designated goal using repulsion forces. The algorithms can be divided into the following three classes: rule-based algorithms, to drive and collect sheep [15–17]; control theoretic approach, to precisely encircle a flock of sheep [18,19]; and learning or optimization-based path planning, to use in obstacle environments [20,21]. These works provide potential solutions to challenges in managing swarm systems, including livestock herding [22,23], crowd control [24] and coordination of large-scale robots and microrobots [25,26].

The current research on the shepherding problem has been based mainly on the prerequisite that steering agents have sufficient sensing capabilities [27], i.e. each agent can recognize sheep agents by their positions and velocities, in conjunction with the positions and velocities of other steering agents, and the position of the goal. However, in practice, robots performing a guiding task may not be able to collect all the expected sensing results from the surrounding environment [28]. Thus, shepherding research incorporating such limitations on the sensing capability of steering agents has been conducted from various perspectives, such as local camera-based observation [29], lack of computation ability or memory [30] and lack of cooperation between multiple steering agents [31].

In addition, in the context of relative bearing measurements, each agent can only measure the relative bearings of its neighbouring agents and not determine the relative distances or proximities [32]. The act of controlling these agents to achieve desired formation patterns is known as bearing-only formation control, which concerns how to accurately coordinate all moving agents [33,34]. Nevertheless, using one set of agents to guide another set of unmanoeuvrable agents using bearing measurements remains a challenging task in swarm control.

This paper presents a bearing-only algorithm for shepherding with limited information. Leveraging the proposed algorithm, we further investigate the minimum amount of information required by the algorithm to guarantee the success of shepherding. The algorithm is inspired by the strategy of a two-stage approach, which divides the movements of a shepherd agent by initially orienting its position relative to its target swarm and then driving the swarm towards the goal [15,20]. The target swarm is one of the swarms that is selected by the shepherd agent among multiple swarms as the target for chasing. Specifically, we first introduce an algorithm using a steering agent, design strategies to allow multiple steering agents to cooperate through reduced collisions and improved efficiency by sharing limited knowledge of bearing measurements (i.e. direction from each position to the estimated centre of each target swarm) and then apply distributed strategies for steering agents to herd multiple swarms. The experiments are conducted for various initial placements with different parameter values for the sheep agents to evaluate the effectiveness and robustness of the proposed algorithm. Finally, we discuss the influence of bearing measurement accuracy and the role of communication between steering agents to understand the requirements of the proposed algorithm.

The remainder of the paper is organized as follows. Section 2 outlines the problem setting of the shepherding problem and the knowledge of shepherd agents in bearing-only measurements. Section 3 presents a step-by-step description of the proposed algorithm. Section 4 shows experimental results to illustrate the functionalities and capabilities of the proposed algorithm under various configurations of parameter values and initial placements, and presents an investigation of the minimum amount of information required.

## 2. Problem settings

The problem of the shepherding task focuses on designing a shepherding algorithm for the steering agents to guide a set of sheep agents into a designated goal region. In this section, we describe the movements of each sheep using an agent-based model to form swarm movements, present the goal setting and design the knowledge of shepherd agents with bearing measurements.

### (a) Sheep model

We consider a situation in which M shepherd agents navigate N sheep agents. It is assumed that, initially, N sheep are in multiple swarms and that M shepherds are positioned at some places around these swarms. The sheep and shepherds are assumed to move dynamically on a two-dimensional plane ${\mathbb{R}}^{2}$ in discrete time. To denote the sets of sheep and shepherds, we use the notation [N]={1,2,…,N} and [M]={1,2,…,M}, respectively. For any i∈[N] and k∈[M], we use $p_{i}(t),u_{i}(t)$ and $q_{k}(t),v_{k}(t)$ to denote the position and velocity, respectively, of the ith sheep and kth shepherd agents, respectively, at time t. The movements of the sheep and shepherd are thus defined as follows:


$$\begin{array}{c} \begin{array}{rl} p_{i}(t+1) & =p_{i}(t)+u_{i}(t),\\ q_{k}(t+1) & =q_{k}(t)+v_{k}(t). \end{array} \end{array}$$

We assume that each sheep and shepherd recognizes other agents within limited sensing ranges with positive values r and $r^{\prime }$, respectively. Therefore, the sets of other sheep and shepherds recognized by the sheep agent i at time t are given by


(2.1) $$\begin{array}{rl} N_{i}(t) & =\{j\in [N]|0<\|p_{i}(t)-p_{j}(t)\|<r\},\\ M_{i}(t) & =\{k\in [M]|0<\|p_{i}(t)-q_{k}(t)\|<r\}, \end{array}$$

respectively.

**Figure 1.**
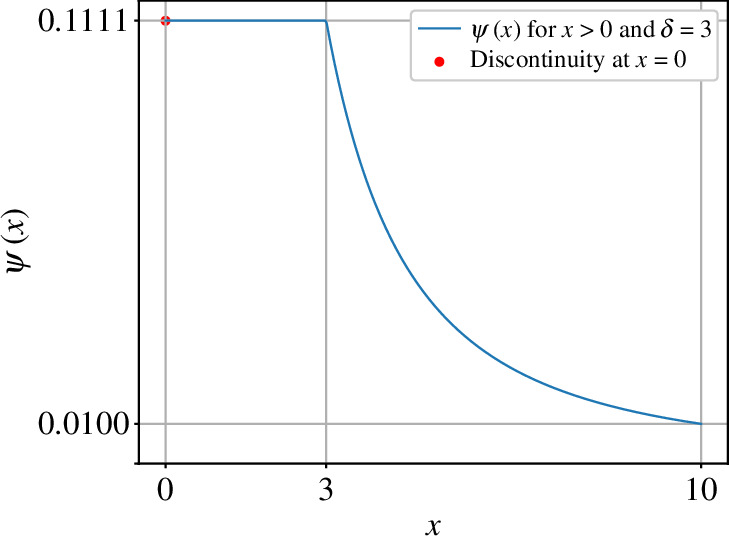
Plotting of the function ψ(x) defined in equation (2.4) when δ=3. The range of x, representing the distance between two agents, is set to x≥0. The value of ψ(x) is 0 at ψ(0), reaches a threshold of $1/\delta^{2}$ when 0<x≤δ and gradually decreases when x≥δ. The values of ψ(x) on the plot are accurate to four decimal places.

Following the convention used for the Boids model [6] and the shepherding problem [11,15,31], the movement of the ith sheep agent at time t is defined by


(2.2) $$\begin{array}{lcr} & u_{i}(t)=c_{1}u_{i1}(t)+c_{2}u_{i2}(t)+c_{3}u_{i3}(t)+c_{4}u_{i4}(t)+c_{5}u_{i5}(t), & \end{array}$$

where $u_{i1}(t)$, $u_{i2}(t)$ and $u_{i3}(t)$ denote the forces of separation, cohesion and alignment, respectively, between sheep; $u_{i4}(t)$ denotes the force of repulsion from the shepherds; and $u_{i5}(t)$ denotes a uniformly distributed random vector representing noise. In addition, $c_{1},c_{2},c_{3},c_{4}$ and $c_{5}$ are positive constants. Specifically, we define the first four vectors as


(2.3) $$\begin{array}{rl} u_{i1}(t) & =-|N_{i}(t){|}^{-1}\sum_{j\in N_{i}(t)}\psi (p_{j}(t)-p_{i}(t)),\\ u_{i2}(t) & =|N_{i}(t){|}^{-1}\sum_{j\in N_{i}(t)}\phi (p_{j}(t)-p_{i}(t)),\\ u_{i3}(t) & =|N_{i}(t){|}^{-1}\sum_{j\in N_{i}(t)}\phi (u_{j}(t-1)),\\ u_{i4}(t) & =-|M_{i}(t){|}^{-1}\sum_{\ell \in M_{i}(t)}\psi (q_{\ell }(t)-p_{i}(t)), \end{array}$$

where ϕ(x)=x/‖x‖ denotes a normalization operator, to represent the direction of a vector, and


(2.4) $$\begin{array}{r} \begin{array}{r} \psi (x)=\left\{ \begin{array}{ll} x/\|x{\|}^{3}, & \text{if }\|x\|\geq \delta ,\\ x/(\|x\|\delta^{2}), & \text{if }0<\|x\|<\delta ,\\ 0, & \text{otherwise}, \end{array}\right. \end{array} \end{array}$$

denotes a potential-like function, to represent the interference caused by the proximity between two agents. We let the constant δ be larger than 1, rather than equal to ‖x‖, to prevent the value of ‖ψ(x)‖ from diverging. One example illustrating the variation of ψ(x) is visualized in figure 1. Among the vectors in equation (2.3), owing to the function ψ(x), both $u_{i1}(t)$ and $u_{i3}(t)$ are calculated based on different scales of distances between each pair of sheep while avoiding collisions when the distances are exceedingly small [35] and avoiding scattering when their distances are exceedingly large. Additionally, we set $u_{2}(t)$ to a time-delay term to allow each agent to attempt to observe and follow the movement of the surrounded sheep.

### (b) Goal

The objective of our shepherding task is to herd all sheep agents into a region called the goal. We let the goal region $G\subset {\mathbb{R}}^{2}$ be a closed disc with centre $g\in {\mathbb{R}}^{2}$ and radius $R_{g}>0$. The value of goal radius $R_{g}$ influences the results of shepherding because the larger the goal radius, the sooner the shepherding is completed. By contrast, the smaller the goal radius, the more time shepherds consume until all sheep are guided into the goal region. Therefore, we set the goal radius $R_{g}$ based on the length of the swarm shape in a relatively stationary state, in which the relative distances between the sheep agents vary over a small range after a few time steps in the absence of interference from the shepherds. The specific settings are introduced in §4.

### (c) Shepherding knowledge

We let a shepherd k herd a swarm using the following knowledge of bearing-only measurements. In the case of a single swarm, all sheep are initially aggregated, and the relative distances between them are limited.

First, we assign each shepherd to observe the other agents under occlusion [29] with bearing measurements. Similar to how we defined equation (2.1), we begin by constructing sets $N_{k}^{\prime }(t)$ and $M_{k}^{\prime }(t)$ and then define $O_{k}^{\prime }(t)=N_{k}^{\prime }(t)\cup M_{k}^{\prime }(t)$ to represent the set containing all other agents within the limited sensing range $r^{\prime }$. For shepherd k, to get a subset $O_{k,\text{occ}}^{\prime }(t)$, we initialize $O_{k,\text{occ}}^{\prime }(t)=\emptyset$ and re-label the indices as $O_{k}^{\prime }(t)=\left\{x_{1}(t),\ldots ,x_{\left|{O_{k}}^{\prime }(t)|\right)}\right\}$ in such a way that $\|x_{1}(t)-q_{k}(t)\|\leq \|x_{2}(t)-q_{k}(t)\|\leq \cdots \leq \|x_{\left|{O_{k}}^{\prime }(t)\right|}(t)-q_{k}(t)\|$. For each $\iota =1,\ldots ,|O_{k}^{\prime }(t)|$, we sequentially join index ι to set $O_{k,\text{occ}}^{\prime }(t)$ if and only if the angular difference from the other agent in $O_{k}^{\prime }(t)$ is larger than a constant $\theta_{\text{occ}}$, which is $|∠\left(x_{\iota }(t)-q_{k}(t),x_{\upsilon }(t)-q_{k}(t)\right)|>\theta_{\text{occ}}$ for any $\upsilon \in O_{k,\text{occ}}^{\prime }(t)$. We then partition $O_{k,\text{occ}}^{\prime }(t)$ in terms of sheep and shepherds to update the sets $N_{k}^{\prime }(t)$ and $M_{k}^{\prime }(t)$, respectively.

For vectors x,y and z, we define $\Theta_{x}(y,z)\in \left[-\pi ,\pi \right)$ by


$$\begin{array}{c} \Theta_{x}(y,z)=∠(z-x,y-x) \end{array}$$

to denote the angle between the vectors z−x and y−x. In this study, the range of any angle is defined to be [−π,π), wherein a negative value indicates clockwise rotation (right) and a positive value indicates counterclockwise rotation (left), to distinguish the right and left directions.

**Figure 2.**
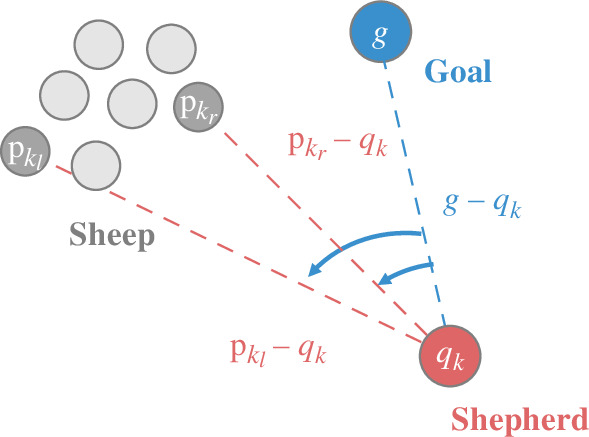
Illustration depicting how shepherd k observes the directions to the left and right positions (dark grey dots) of the swarm boundary relative to the goal region according to equation (2.5). Red dot: shepherd; grey dots: sheep; blue dot: goal centre. Blue dashed line: direction from shepherd to goal; red dashed lines: directions from shepherd to two sheep; angles shown as blue curves between the blue line and two red lines: minimum and maximum values. Time t is omitted here and in subsequent illustrations.

Then, from the position of shepherd k, the positions of the sheep on the right and left sides of the swarm are given by


(2.5) $$\begin{array}{lcr} & \begin{array}{ll} p_{k_{r}}(t) & ={\mathrm{arg min}}_{p\in \{p_{i}(t){\}}_{i\in N_{k}^{\prime }(t)}}\;\Theta_{q_{k}(t)}(g,p),\\ p_{k_{l}}(t) & ={\mathrm{arg max}}_{p\in \{p_{i}(t){\}}_{i\in N_{k}^{\prime }(t)}}\;\Theta_{q_{k}(t)}(g,p), \end{array} & \end{array}$$

while shepherd k knows only the direction from itself to these two positions. Figure 2 is a visualization how the angles and positions are calculated. Subsequently, we assume that shepherd k has the following three vectors at time t:


(2.6) $$\begin{array}{lcr} & Q_{k}(t)=\{\phi (p_{k_{r}}(t)-q_{k}(t)),\phi (p_{k_{l}}(t)-q_{k}(t)),\phi (g-q_{k}(t))\} & \end{array}$$

instead of the following positions: $N_{k}^{\prime }(t)$, $M_{k}^{\prime }(t)$ and g.

We then average the vectors to the left and right sides to obtain a vector to the estimated swarm centre, denoted as $c_{k}(t)$, such that $\phi (c_{k}(t)-q_{k}(t))=\phi (\phi (p_{k_{r}}(t)-q_{k}(t))+\phi (p_{k_{l}}(t)-q_{k}(t)))$. The angle between the direction from shepherd k to the estimated swarm centre $c_{k}(t)$ and the direction from shepherd k to goal g is then denoted as $\Theta_{q_{k}(t)}(c_{k}(t),g)$ based on knowledge $Q_{k}(t)$ defined in equation (2.6). Additionally, we assume that shepherd k can memorize only $Q_{k}(t)$ at each time step and, therefore, cannot estimate the relative distance to any agent based on the change in angle over time. Because the shepherd cannot measure how far it has moved, we assign a fixed size for the velocity of each shepherd, denoted $\|v_{k}(t)\|=d$, where d is a positive constant.

## 3. Proposed algorithm

We first introduce the proposed algorithm based on a single shepherd (M=1) herding a single swarm. Afterwards, we extend the algorithm to allow for multiple shepherds herding multiple swarms through cooperation between shepherds and strategies for recognizing swarms. The overall concept in constructing the algorithm is for each shepherd to perceive swarms as one or multiple masses based on the angular difference to individuals and guide each mass sequentially using the orientation and driving stages. The movement of shepherds relative to the mass is simplified by determining whether to move in the direction of the left or right boundary using reasonable rules. The limited angular information to individuals is sufficient to avoid disturbance from shepherds to individuals inside the swarms.

### (a) Single-shepherd herding of one swarm

**Figure 3.**
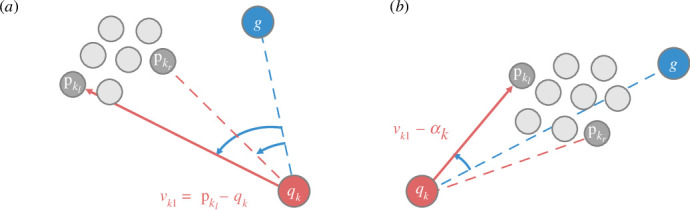
Illustration showing a shepherd herding a swarm to the goal region. The shepherding process is divided into two stages: orientation (*a*) and driving (*b*). Red solid line with arrow: movement direction of shepherd. During the orientation stage, a shepherd moves towards the sheep $p_{k_{l}}(t)$ or $p_{k_{r}}(t)$ according to the unit vector $\alpha_{k}(t)$ determined by comparing angles (indicated by blue) as in equation (3.2). During the driving stage, a shepherd continuously moves towards one sheep until the angle indicated by a blue curve is small enough.

In this part of the study, we use a reduction method that allows the shepherd to regard each swarm as a mass rather than a set of individuals. Our algorithm includes two stages, i.e. orienting behind the swarm relative to the goal and driving the swarm towards the goal by switching between the two directions of the swarm borders. Compared to previous two-staged algorithms in shepherding [15,18,20], the proposed algorithm only requires the shepherd agent to have bearing measurements, and the target swarm is modelled using a nonlinear agent-based model as denoted in equation (2.2). Specifically, we judge these two stages by examining angle $\Theta_{q_{k}(t)}(c_{k}(t),g)$ and comparing it against a threshold $\theta_{\text{orient}}$ using the expression


(3.1) $$|\Theta_{q_{k}(t)}(c_{k}(t),g)|<\theta_{\text{orient}},$$

where a result of ‘false’ indicates orientation, and a result of ‘true’ indicates driving; $\theta_{\text{orient}}$ is chosen to be sufficiently small enough to prevent judging two separate swarms as one, yet large enough to ensure that the shepherd does not mistakenly consider one swarm as multiple masses, particularly when the shepherd is close to the swarm. Figure 3 illustrates the circumstances of each of the two stages.

Let us first introduce notation commonly used in both stages. Depending on which side has the larger angle to the goal direction, we define unit vectors $\alpha_{k}(t)$ and $\alpha_{k}^{\prime }(t)$ as


(3.2) $$\begin{array}{r} \alpha_{k}(t)=\left\{ \begin{array}{ll} \phi (p_{k_{r}}(t)-q_{k}(t)), & \text{if }|\Theta_{q_{k}(t)}(p_{k_{r}}(t),g)|\geq |\Theta_{q_{k}(t)}(p_{k_{l}}(t),g)|,\\ \phi (p_{k_{l}}(t)-q_{k}(t)), & \text{otherwise}, \end{array}\right. \end{array}$$

and


(3.3) $$\begin{array}{r} \alpha_{k}^{\prime }(t)=\left\{ \begin{array}{ll} \phi (p_{k_{r}}(t)-q_{k}(t)), & \text{if }|\Theta_{q_{k}(t)}(p_{k_{r}}(t),g)|<|\Theta_{q_{k}(t)}(p_{k_{l}}(t),g)|,\\ \phi (p_{k_{l}}(t)-q_{k}(t)), & \text{otherwise}, \end{array}\right. \end{array}$$

where $\alpha_{k}(t)$ represents the direction where the angle relative to the goal direction is larger compared to the other direction, while $\alpha_{k}^{\prime }(t)$ represents the other direction. We then let a unit vector $v_{k1}(t)$ represent the primary direction of movement for the shepherd. The vector $v_{k1}(t)$ is assigned a value equal to either $\alpha_{k}(t)$ or $\alpha_{k}^{\prime }(t)$, which is determined by the following algorithm. In addition, the shepherd receives repulsion from the direction of the estimated swarm centre $c_{k}(t)$ and goal g. The velocity of shepherd k is then derived to be


(3.4) $$\begin{array}{r} v_{k}(t)=d_{q}\phi (d_{1}R(\theta_{1},v_{k1}(t))+d_{2}\phi (q_{k}(t)-c_{k}(t))+d_{3}\phi (q_{k}(t)-g)) \end{array},$$

where $d_{1}$, $d_{2}$ and $d_{3}$ are positive constants, of which $d_{1}$ is larger than the others. In addition, $R(\theta_{1},v_{k1}(t))$ is an operator defined as


(3.5) $$\begin{array}{r} R(\theta_{1},v_{k1}(t))=\left\{ \begin{array}{ll} \left[\begin{array}{cc} \cos\theta_{1} & \sin\theta_{1}\\ -\sin\theta_{1} & \cos\theta_{1} \end{array}\right]v_{k1}(t), & \text{if }v_{k1}(t)=\phi (p_{k_{r}}(t)-q_{k}(t)),\\ \left[\begin{array}{cc} \cos\theta_{1} & -\sin\theta_{1}\\ \sin\theta_{1} & \cos\theta_{1} \end{array}\right]v_{k1}(t), & \text{otherwise}, \end{array}\right. \end{array}$$

to rotate vector $v_{k1}(t)$ by a non-negative angle $\theta_{1}$. The direction of rotation depends on which side $v_{k1}(t)$ lies: if on the right side, then rotate right, and if on the left side, then rotate left. Equation (3.5) is used to rotate the movement of the shepherd away from the swarm to avoid collision risk.

#### (i) Orientation stage

Given that the shepherd cannot measure the distance to the other agents, moving directly towards the swarm may result in collisions. In cases wherein the angle $|\Theta_{q_{k}(t)}(c_{k}(t),g)|$ between the direction from shepherd k to the swarm and the direction from shepherd k to goal g is very large, as indicated by a result of ‘false’ in equation (3.1), the shepherd needs to orient behind the swarm relative to the goal to reduce the angular difference $|\Theta_{q_{k}(t)}(c_{k}(t),g)|$.

Specifically, we choose the direction having the larger angular difference between the left and right sides of the swarm and denote it as $v_{k1}(t)$, i.e. we let $v_{k1}(t)=\alpha_{k}(t)$. Throughout the orientation stage, as the shepherd moves to one side, the angle on that side increases, and thus the shepherd is expected to continuously move to the same side of the swarm based on the comparison of angles in equation (3.2).

#### (ii) Driving stage

After completing the orientation stage, which is indicated by a result of ‘true’ in equation (3.1), the shepherd begins to drive the swarm by alternately switching between the right and left sides. Based on $\alpha_{k}(t)$ defined in equation (3.2), we define another unit vector as


(3.6) $$\begin{array}{r} {\tilde{\alpha }}_{k}(t)=\left\{ \begin{array}{ll} \phi (p_{k_{r}}(t)-q_{k}(t)), & \text{if }\alpha_{k}(t-1)=\phi (p_{k_{r}}(t-1)-q_{k}(t-1)),\\ \phi (p_{k_{l}}(t)-q_{k}(t)), & \text{otherwise}, \end{array}\right. \end{array}$$

which remains at the same right or left side as that in the previous time step t−1 when t>0 and keeps the same value as ${\tilde{\alpha }}_{k}(t)=\alpha_{k}(t)$ when t=0. Then, similar to how we defined equation (3.3), we denote a unit vector on the other side as ${\tilde{\alpha }}_{k}^{\prime }(t)$. We then let $v_{k1}(t)$ be


(3.7) $$\begin{array}{r} \begin{array}{r} v_{k1}(t)=\left\{ \begin{array}{ll} {\tilde{\alpha }}_{k}^{\prime }(t), & \text{if }\text{sgn}∠\left({\tilde{\alpha }}_{k}(t),{\tilde{\alpha }}_{k}^{\prime }(t)\right)=\text{sgn}∠\left({\tilde{\alpha }}_{k}(t),g-q_{k}(t)\right)\\ & \text{ and }\left|∠\left({\tilde{\alpha }}_{k}(t),g-q_{k}(t)\right)\right|<\theta_{\text{drive}},\\ {\tilde{\alpha }}_{k}(t), & \text{otherwise}, \end{array}\right. \end{array} \end{array}$$

where the operator sgn indicates whether a value is positive or negative, and $\theta_{\text{drive}}$ is a positive constant. At each time step, $v_{k1}(t)$ is adjusted to remain on the same side as that in the previous time step unless it is already at the edge of that side as determined by the angle conditions in equation (3.7). Throughout the driving stage, the shepherd is expected to move to one of the right or left sides for a while, then switch to the other side and repeat this switching movement.

### (b) Multi-shepherd herding of one swarm

In this part of the study, we increase the number of shepherds to perform the shepherding task more effectively. To avoid moving repeatedly between multiple shepherds, we design a strategy for cooperation between two shepherds, denoted as k,ℓ, and subsequently apply it to more shepherds.

We allow communication between the shepherds because relying solely on the direction from shepherd k to shepherd ℓ is not sufficient for accessing their orientation relative to the swarm for cooperation. Specifically, for shepherd k, we define the shared information as the direction from another shepherd ℓ to its estimated swarm centre $c_{\ell }(t)$. This additional knowledge is denoted


(3.8) $$\begin{array}{lcr} & \begin{array}{r} Q_{k\ell }(t)=\{\phi (c_{\ell }(t)-q_{\ell }(t))\}. \end{array} & \end{array}$$

Subsequently, we update the knowledge of shepherd k in equation (2.6) to obtain the orientation of all the other shepherds as


(3.9) $$\begin{array}{r} {\bar{Q}}_{k}(t)=Q_{k}(t)\cup \underset{\ell \in M_{k}^{\prime }(t)}{⋃}Q_{k\ell }(t). \end{array}$$

**Figure 4.**
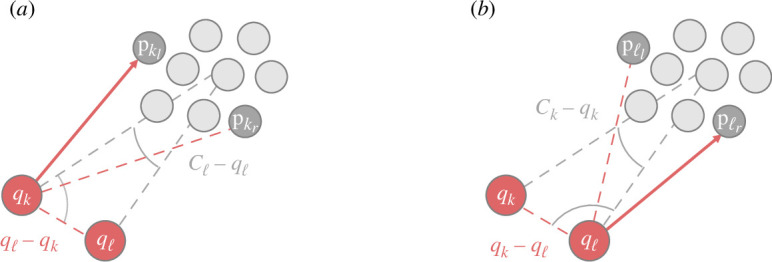
Illustration depicting two shepherds cooperating to herd a swarm. Knowledge and next movements of shepherds k (*a*) and ℓ (*b*) are shown. Grey dashed lines: directions from each shepherd to their respective estimated centres, which are shared between both shepherds; angles shown as grey curves: for calculation of their next movements, in accordance with algorithm 1; red solid lines with arrows: movement of shepherd k to its left and shepherd ℓ to its right at next time step.

Based on the updated knowledge ${\bar{Q}}_{k}(t)$, we extend the proposed algorithm to allow for multiple shepherds herding one swarm. Each shepherd independently decides its current stage. During the orientation stage, each shepherd determines its direction without considering the presence of other shepherds. During the driving stage, the movement of each shepherd is adjusted to avoid repeated movements with other shepherds. Specifically, we propose algorithm 1 to modify $v_{k1}(t)$, where each shepherd can estimate the number of shepherds on its potential path to the right or left and choose to move to the side that has fewer shepherds. This estimation relies on comparing angles between specific directions observed by each shepherd and directions shared among them, as denoted by equation (3.9). Specifically, for shepherds k and ℓ, the angle compared with $\theta_{n1}$ evaluates whether $q_{k}(t)$ and $q_{\ell }(t)$ have similar directions to the swarm centre, while the angle compared with $\theta_{n2}$ evaluates whether $q_{\ell }(t)$ and the swarm centre have similar directions to $q_{k}(t)$. If both conditions are met, shepherd ℓ is judged to be near the potential movement path of shepherd k. Shepherd k then determines whether that shepherd ℓ is on the left or right path; $\theta_{n1}$ and $\theta_{n2}$ are fixed values. Additionally, although the vectors $\phi (c_{k}(t)-q_{k}(t))$ and $\phi (c_{\ell }(t)-q_{\ell }(t))$ are pointing to different estimated swarm centres, we consider the error to be negligible. Figure 4 illustrates the shared information and movements of two shepherds during the driving stage.


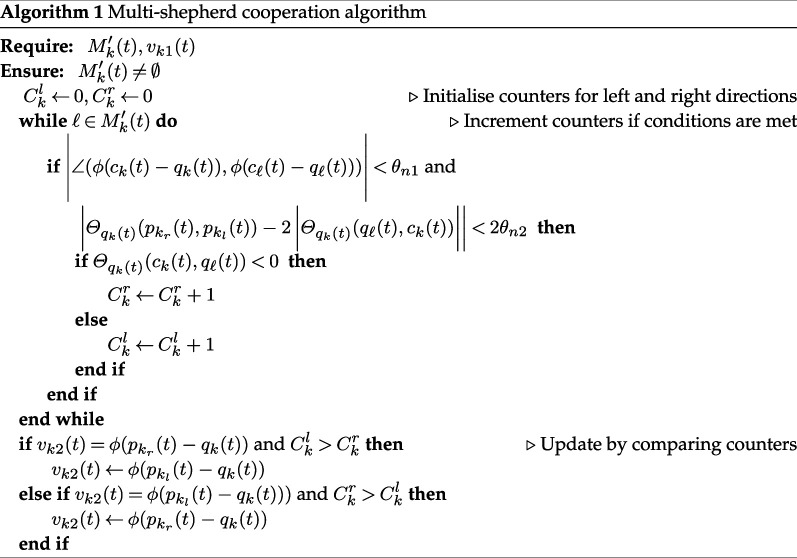


### (c) Multi-shepherd herding of multiple swarms

In this part of the study, we place multiple swarms separately in their initial placements. Each shepherd lacks knowledge of the number of swarms based on its knowledge ${\bar{Q}}_{k}(t)$ defined in equation (3.9). Instead, it observes these swarms as subswarms by comparing the angles of interval between sheep with a threshold $\theta_{n}$. Therefore, the set of sheep observed under occlusion by shepherd k, denoted as $N_{k}^{\prime }(t)$, is partitioned into multiple subswarms. Specifically, we partition set $N_{k}^{\prime }(t)$ to each subswarm τ as $N_{k}^{\prime }(t)={⋃}_{\tau }N_{k\tau }^{\prime }(t)$ in such a way that any pair of sheep $i\in N_{k\tau }^{\prime }(t)$ and $j\in N_{k\tau^{\prime }}^{\prime }(t)$ satisfies $|\Theta_{q_{k}(t)}(p_{i}(t),p_{j}(t))|>\theta_{n}$ if and only if $\tau \neq \tau^{\prime }$.

**Figure 5.**
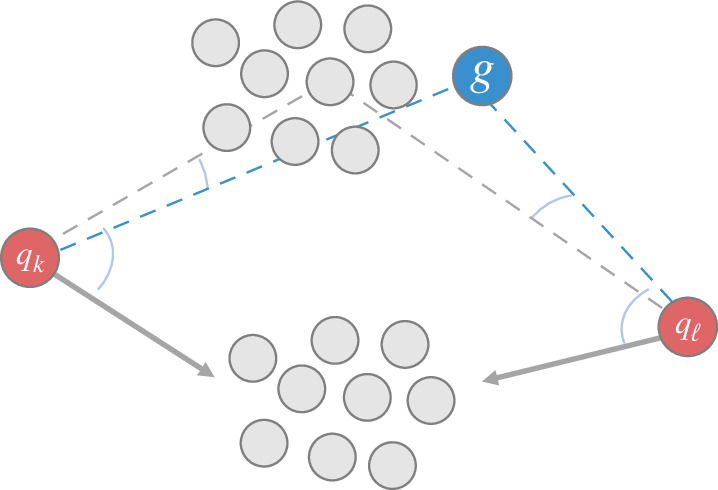
Illustration depicting two shepherds herding two swarms by targeting a subswarm. Angles shown as blue curves: for comparison between each shepherd to choose a subswarm having a larger angle; grey solid lines with arrows: the subswarm targeted by both shepherds (herein, both are towards the bottom swarm) based on equation (3.10).

Cooperation between the shepherds can be established by letting each shepherd sequentially target a specific subswarm rather than all subswarms. We first label the subswarm that has the largest absolute angle between the direction to its estimated centre and the direction to the goal as


(3.10) $$\begin{array}{rl} N_{k}^{\prime \text{max}}(t) & =\max_{\tau }\left|\Theta_{q_{k}(t)}(c_{k\tau }(t),g)\right| \end{array},$$

where $c_{k\tau }(t)$ represents the estimated centre of subswarm τ observed by shepherd k. We then let the shepherd execute the algorithm by observing sheep in the subset $N_{k}^{\prime \text{max}}(t)$ rather than $N_{k}^{\prime }(t)$. Following this strategy, each shepherd chooses and herds its target subswarm $N_{k}^{\prime \text{max}}(t)$ at each time step until all the swarms are inside the goal region. Figure 5 illustrates how two shepherds choose their target subswarms.

In addition, each shepherd is unaware of whether other shepherds are targeting the same subswarm and needs only to estimate if the others are on its potential path, in accordance with algorithm 1, to cooperate.

Summary: through the descriptions presented earlier, we have proposed an algorithm by which varying numbers of shepherds can cooperatively herd multiple swarms in different distributions. The shepherds follow the final design of the algorithm regardless of their placements in the experiments.

## 4. Experiments

Our aim in the experiments was to test whether shepherding can be successful and to determine the minimum amount of information required for the proposed algorithm. For the experiment, we first assign values to the parameters of the sheep model and shepherding algorithm, set-up the initial placements of the experiments and design metrics to evaluate the performance of the shepherding algorithm. We present simulation results that illustrate the trajectory and time-series variations for a single trial and an evaluation across multiple trials. Finally, we reduce the angular accuracy of the bearing measurements and remove communication between the shepherds to conduct experiments to measure the information required to successfully accomplish the shepherding task.

The experiments were conducted on hardware consisting of Ubuntu 22.04 LTS running on an Intel(R) Xeon(R) Gold 6330 CPU @ 2.00 GHz (28 cores, 56 threads) in a dual-socket configuration, with 755 GB of RAM.

### (a) Parameter values

In this part of the study, we select different parameter values and placements for the sheep agents and observe their movements under these settings to determine an appropriate goal radius for shepherding. Additionally, we assign the parameter values for the shepherding algorithm and explain their rationales.

#### (i) Sheep agents

Because different parameter values for the same sheep model can result in a variety of behaviours, we conduct experiments under several sets of parameter values $c_{1},c_{2},c_{3},c_{4},c_{5}$ and compare their levels of performance. Here, based on the movement characteristics of swarm systems [36], we present three sets of parameter values for the sheep agents, as follows:


(4.1) $$\begin{array}{lcr} & \begin{array}{rl} c_{1} & =200,c_{2}=0.2,c_{3}=0.2,c_{4}=1000,c_{5}=0.1,r=60,\\ c_{1} & =250,c_{2}=0.15,c_{3}=0.2,c_{4}=1200,c_{5}=0.2,r=60,\\ c_{1} & =200,c_{2}=0.2,c_{3}=0.25,c_{4}=800,c_{5}=0.05,r=60, \end{array} & \end{array}$$

where we classify the first set as the baseline; the second set as sensitive, owing to its larger separation $c_{1}$, smaller cohesion $c_{2}$, tendency to separate under larger repulsion $c_{4}$ and larger noise $c_{5}$; and the third set as insensitive, owing to its tendency to align with the others under larger alignment $c_{3}$, smaller repulsion $c_{4}$ and smaller noise $c_{5}$. Additionally, we set δ=3 in equation (2.4).

#### (ii) Goal radius

The size of the goal radius greatly influences the performance of the shepherding algorithm. If the goal radius is much smaller than the radius of the swarm, the shepherds are likely to keep circling the goal point and its periphery without completing the task successfully. Alternatively, if the goal radius is set to be exceedingly large, the shepherds will easily complete the task, leaving us unable to assess the performance.

Therefore, we determine the goal radius by observing the shape of the swarm when it is relatively stationary without any shepherds or obstacles. Specifically, we measure the length of the swarm shape in terms of the maximum distance between agents, which is $x_{s}(t)=\max_{i,j\in N(t)}\;\left\|p_{i}(t)-p_{j}(t)\right\|$. Then, by observing the time-series variation of $x_{s}(t)$, we determine that the swarm is stationary when $x_{s}(t)$ has little variation over time, i.e. if it satisfies $(1-k_{s})x_{s}(t_{s}+1)<x_{s}(t_{s})<\left(1+k_{s})x_{s}(t_{s}+1\right)$ with $k_{s}=0.02$ when $t\geq t_{s}$. We then define the goal radius $R_{g}=k_{g}x_{s}\left(t_{s}\right)$ with the coefficient $k_{g}=0.8$. This procedure is followed to ensure a common goal radius for the subsequent experiments conducted under different sets of parameter values for the same placements, as outlined in equation (4.1). When calculating the goal radius for multiple swarms, we consider that the shepherds must be able to collect all the swarms into the goal region. We first calculate the expected goal region for each swarm, then summarize the approximated goal radius for all sheep agents in these swarms.

Additionally, given that shepherds rely solely on bearing measurements and cannot independently judge whether all the sheep are inside the goal region, the completion of a shepherding task is determined externally and uniformly for all the shepherds.

#### (iii) Shepherd agents

For the parameter values of shepherds, we fix the magnitude of velocity for each shepherd at $d_{q}=2$. This ensures that the velocity of the shepherds is moderately higher than that of the sheep agents, as determined by the parameter values given in equation (4.1). Additionally, regarding other coefficients appearing in equation (3.4), we set $d_{1}$ to be much larger than $d_{2}\;and\;d_{3}$, using $d_{1}=5$, $d_{2}=1$ and $d_{3}=1$ to make $v_{k1}(t)$ the primary movement. We assign the sensing range $r^{\prime }=300$ to ensure that the range is sufficiently large for sensing other agents. We then assign the angle thresholds for each shepherd as $\theta_{\text{occ}}=\pi /60$, $\theta_{1}=\pi /9$, $\theta_{\text{orient}}=\pi /3$, $\theta_{\text{drive}}=\pi /18$, $\theta_{\text{n1}}=\pi /4$, $\theta_{\text{n2}}=\pi /2$ and $\theta_{n}=\pi /6$. These angles are assigned appropriate values based on the following rationales: angle $\theta_{\text{occ}}$ is set to a small value to imitate observation under occlusion; angle $\theta_{1}$ is moderately adjusted to avoid collisions between the shepherds and swarms; angle $\theta_{\text{orient}}$ is limited to no more than π/2 to determine the orientation or driving stage; angle $\theta_{\text{drive}}$ is appropriately small to allow complete driving on one side before switching to the other side; angles $\theta_{\text{n1}},\theta_{\text{n2}}$ are chosen reasonably to determine whether there are other shepherds on their paths; and angle $\theta_{n}$ is appropriately small to ensure correct recognition of subswarms.

### (b) Initial placements

For the next part of the experiment, we design three initial placements of the sheep and shepherd agents where N sheep are distributed into n different swarms. Specifically, each swarm, denoted by σ, consists of $N_{\sigma }$ sheep that are randomly placed on a disc centred at each origin with an initial radius of $R_{s\sigma }$ when t=0. We denote the numbers and radii of multiple swarms as

— $N_{1}=30,R_{\text{s1}}=40$,
— $N_{1}=30,R_{\text{s1}}=40$ and $N_{2}=50,R_{\text{s2}}=60$,
— $N_{1}=30,R_{\text{s1}}=40$, $N_{2}=30,R_{\text{s2}}=40$ and $N_{3}=50,R_{\text{s3}}=60$.

**Figure 6.**
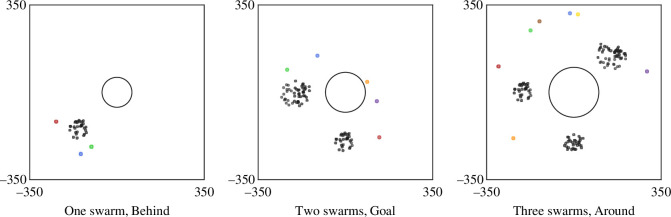
Initial placements of swarms, shepherds and goal. Positions of sheep vary from one to three swarms around the goal. Numbers of shepherds are M=3, M=5 and M=7, respectively. Positions of shepherds are categorized as behind the swarms, near the goal or around the swarms. Shepherds are behind the swarms if the shepherds are farther from the goal than the swarms, with similar directions to the goal; shepherds are near the goal if the shepherds are close to the goal centre; shepherds are around the swarms if the shepherds are farther from the goal than the swarms, with directions to the goal coming from all around.

We then set the placements between these swarms and between the swarms and the goal, and position the shepherds at various positions to the swarms relative to the goal, such as behind the swarms, near the goal and around the swarms, as illustrated in figure 6.

### (c) Evaluation metrics

We design the following two metrics to evaluate the effectiveness and stability of the proposed algorithm in the shepherding task. The first metric measures progression in individual trials, whereas the second measures performance across multiple trials.

Time-series variations in distances over time: This metric calculates the distances from the sheep and shepherd agents to the goal during each trial. In successful trials, we observe that the distance from each sheep agent i to the goal, $\left|p_{i}(t)-g\right|$ decreases from an initial value to a value below the goal radius $R_{g}$. We record the mean value and the upper and lower intervals for the sheep agents. Similarly, we denote the distance from shepherd k to the goal as $\left|q_{k}(t)-g\right|$. The mean value for the shepherds usually follows the values for the sheep because the shepherds herd the sheep to the goal region.

Consumed time: This metric measures the overall performance of the shepherds across multiple trials. The total time consumed in all trials is counted and used to draw box plots that visualize the experiment results. Low consumed time and small variation between trials indicate effective and robust shepherding.

### (d) Experiment results

**Figure 7.**
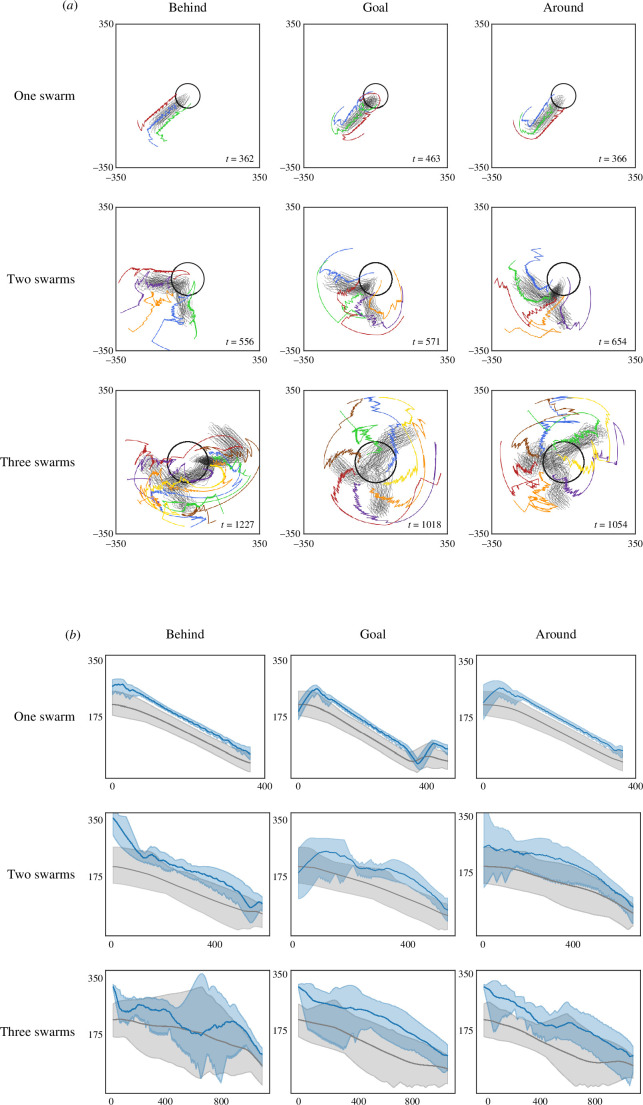
Herding one to three swarms for three types of initial shepherd placements, with baseline set of sheep-model parameter values. Numbers of shepherds are M=3, M=5 and M=7 for the case of one, two and three swarms, respectively. (*a*) The trajectories with same initial placements as in figure 6. (*b*) The time-series variations in distance from shepherds and sheep to the goal. Blue line and interval: shepherds; grey line and interval: sheep.

We conduct experiments in C=20 trials with an upper time limit T for each placement. The shepherding succeeds only when the consumed time is shorter than the upper limit T. Specifically, we set T=3000 to ensure sufficient time steps to successfully complete the shepherding task. We conduct simulation experiments for three placements using the appropriate number of shepherds and the baseline parameter values for sheep. For the tasks of shepherding a swarm, two swarms and three swarms, we show the trajectories in figure 7*a* and numerically illustrate the shepherding process in figure 7*b* by displaying the time-series variation in the distances of the sheep and shepherd agents to the goal for a random trial.

From the trajectories, we observe that the movement of each shepherd is practically divided into two stages and repeated several times, especially in the cases of shepherding three swarms, which aligns with the proposed algorithm that includes the orientation and driving stages. When multiple shepherds herd the same swarm, the movements at each time step indicate that the shepherds can recognize neighbouring shepherds and avoid converging towards each other. This phenomenon naturally results in the shepherds dynamically encircling the target swarm and collectively driving it to the goal region. Furthermore, when herding multiple swarms, each shepherd can estimate the orientation of other shepherds relative to its target subswarm and sequentially drive the swarms without duplicating movements with the other shepherds.

**Figure 8.**
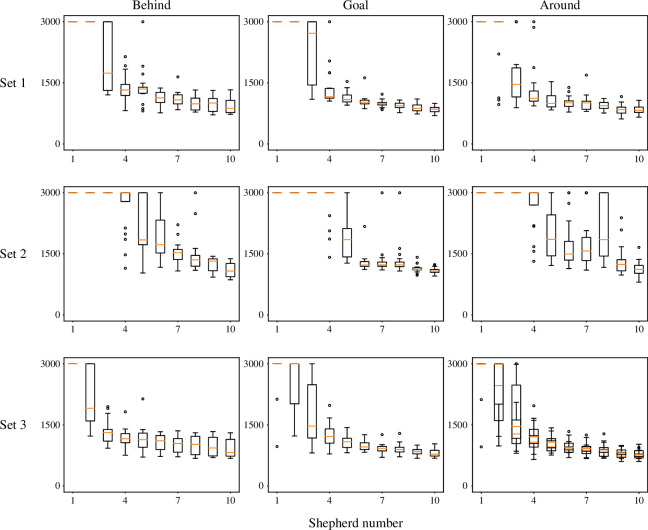
Box plots of time consumed shepherding three swarms with respect to number of shepherd agents, for three sets of sheep-model parameter values. Number M of shepherds varies from 1 to 10.

We then evaluate the shepherding performance given the initial placements described earlier and using three sets of parameter values for the sheep, with the number of shepherds increasing from 1 to 10. The results are presented as changes in consumed time across C trials. In figure 8, to avoid redundancy, we present only the results for shepherding three swarms. We observe that while the shepherds struggle to succeed with shepherding when their numbers are low, success rates increase and consumed time decreases as the number of shepherds increases, eventually reaching a 100% success rate and gradually decreasing the consumed time, which demonstrates the significance of communication. We then compare the differences in shepherding results among the three sets of parameter values. We note that shepherding sensitive sheep tends to fail, whereas shepherding insensitive sheep tends to succeed and consume less time. Nevertheless, the shepherding results generally remain stable regardless of changes in the parameter values for the sheep agents.

Furthermore, we investigate the minimum amount of information, in terms of angular accuracy and communication, required for shepherding. We determine that moderate angular accuracy and limited communication between shepherds are necessary for our bearing-only algorithm.

**Figure 9.**
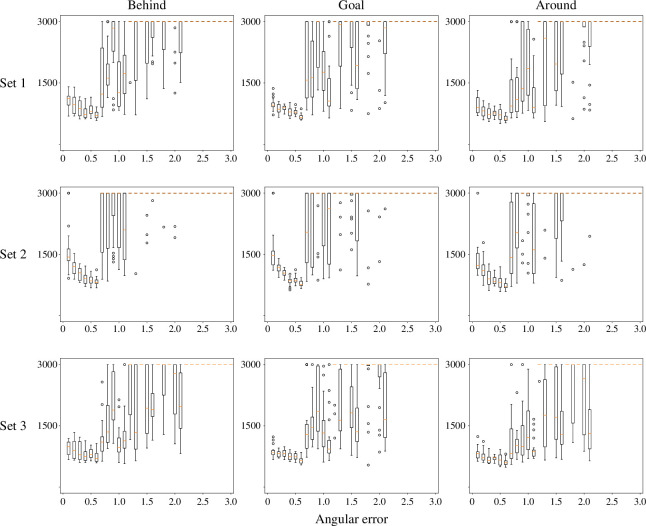
Box plots of time consumed shepherding three swarms with respect to angular error, for three sets of sheep-model parameter values. Number of shepherds is set to M=7, and angular error increases from 0 by 0.1 to 3.

**Figure 10.**
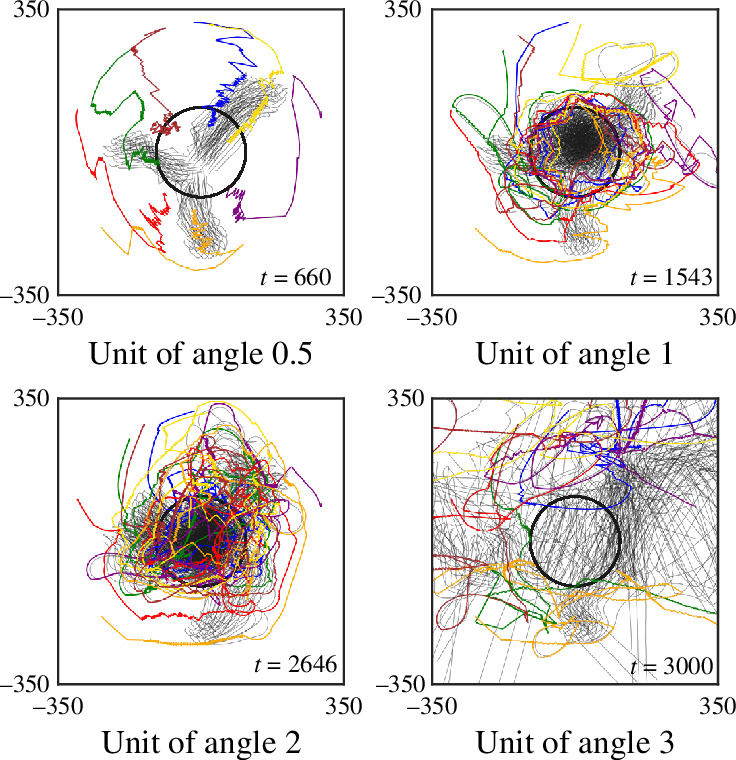
Trajectories of shepherding three swarms as unit of angle increases from 0.5 to 1, 2 and 3, for baseline set of sheep-model parameter values. Initial placements are the same as in figure 6.

With regard to angular accuracy, we increase the error for any angle measured by rounding the value of each angle down to the nearest multiple of a unit, starting from a baseline with no error, ranging from 0.1 to 3, with increments of 0.1, as used in bearing measurements outlined in equation (2.6). In the experiments, we assign M=7 to be the number of shepherds that are to herd three swarms, which is sufficient for success with no angular error. The results are shown in figure 9. We observe that when the error is small, the consumed time does not change significantly and may even (approximately) approach 0.5. We believe that this phenomenon occurs because, under the assumption that there is no error in measuring the angles, the movements of the shepherds result in unnecessary reactions to minor changes in the angle. This oscillation decreases as the error increases. However, as the error continuously increases, the consumed time begins to fluctuate, and the success rate significantly decreases. Trajectories with increasing angular error are illustrated in figure 10. The trajectory varies depending on whether the shepherding succeeds or fails. In successful trials, shepherds usually herd the swarms while maintaining the shape of the swarm until all the sheep agents reach the goal region. On the other hand, in failed trials, the shepherds gradually lose precise control of the swarms as the angular error increases. With larger errors, the shape of the swarm may exceed the size of the goal region even if the shepherds continue circling the swarm to herd it into the goal region. With even larger errors, the swarm may become completely fragmented and scattered by the shepherds.

**Figure 11.**
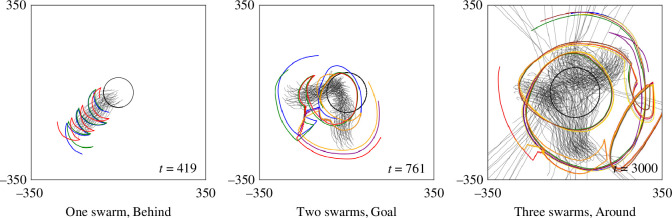
Trajectories of shepherding one to three swarms with no communication between shepherds, for baseline set of sheep-model parameter values. Initial placements, as well as the number of sheep and shepherd agents, are the same as in figure 6, and method is compared with that for figure 7*a*.

With regard to communication, the proposed algorithm requires only the information $Q_{k\ell }(t)$ given in equation (3.8). If we attempt to remove the only communication between each pair of shepherds, there would be no cooperation among the shepherds, potentially leading to collisions and overlapping movements. An example of shepherding without communication is illustrated in figure 11, where shepherd number M=3 for herding one swarm, M=5 for herding two swarms and M=7 for herding three swarms. The trajectories of shepherds become repetitive owing to the lack of communication, which prevent accounting for the presence of other shepherds, thus failing to differentiate their movements. As the effectiveness does not improve with an increasing number of shepherds, this approach takes longer to complete and is more likely to fail when multiple swarms exist.

## 5. Conclusion

In this study, we introduce a bearing-only algorithm for coordinating multiple shepherd agents to herd swarms to a common goal region and investigate the minimum information required to complete this shepherding task. Initially, we employ a reduction method that emphasizes the collective movements of entire swarms rather than individual sheep agents. Each shepherd agent is enabled to sense the orientation of the two boundaries of each swarm and recognize multiple swarms based on angular differences. Subsequently, we propose a generalized shepherding algorithm that does not require knowledge of the exact swarm model or individual sheep agents. Additionally, we devise methods for shepherds to select target swarms and cooperate in the driving stage by confirming their relative orientations to the swarms. Experiments are conducted to evaluate performance under different placements and parameter values, demonstrating the effectiveness of the proposed algorithm with varying numbers of shepherds. Furthermore, we investigate the roles of angular accuracy and communication among shepherds in shepherding, and the minimum conditions for both types of information required in shepherding.

The theoretical framework provided by the bearing-only algorithm offers crucial insights into swarm dynamics, emphasizing understanding the collective behaviour of swarms and the required information for nonlinear control. This foundation aligns with practical examples of swarm applications across domains such as agriculture and search and rescue, highlighting the transformative potential of swarm systems. Future research will prioritize refining algorithms and fostering interdisciplinary collaboration to advance swarm technology, leveraging advancements in artificial intelligence, sensors and robotics. Bridging theory and application, swarm systems stand poised to revolutionize industries and enhance human well-being, promising innovative solutions to complex challenges and expanding the frontiers of technology.

## Data Availability

Simulation program, initial configurations and sample data can be found at [37].
